# Preanalytical issues related to routine and diagnostic glucose tests: Results from a survey in Spain

**DOI:** 10.11613/BM.2020.010704

**Published:** 2019-12-15

**Authors:** Isabel García-del-Pino, Josep M. Bauça, Carolina Gómez, Andrea Caballero, María Antonia Llopis, Mercedes Ibarz, Débora Martínez, Montserrat Ventura, Itziar Marzana, Juan J. Puente, Marta Segovia, Paloma Salas, Rubén Gómez-Rioja

**Affiliations:** 1 Spanish Society of Laboratory Medicine, Extra-analytical Quality Commission, Barcelona, Spain; 2 Area Laboratory, A Coruña University Hospital Complex, A Coruña, Spain; 3 Department of Laboratory Medicine, Son Espases University Hospital, Palma, Balearic Islands, Spain; 4 Department of Clinical Analysis and Biochemistry, Laboratori Clínic Metropolitana Nord, Germans Trias i Pujol Hospital, Badalona, Spain; 5 Preanalytic Area, Department of Clinical Biochemistry, Vall d’Hebron Hospital, Barcelona, Spain; 6 Clinical Laboratories Corporate Coordination, Catalan Health Institute, Barcelona, Spain; 7 Department of Laboratory Medicine, Arnau de Vilanova University Hospital, IRBLleida, Lleida, Spain; 8 Department of Laboratory Medicine, University of Navarra Clinic, Madrid, Spain; 9 External Quality Assurance Programmes, Spanish Society of Laboratory Medicine, Barcelona, Spain; 10 Department of Laboratory, Cruces University Hospital, Barakaldo, Bizkaia, Spain; 11 Department of Biochemistry, ‘Lozano Blesa’ University Clinical Hospital, Zaragoza, Spain; 12 Department of Laboratory Medicine, La Paz-Cantoblanco-Carlos III University Hospital, Madrid, Spain; 13 Department manager of Preanalytical and Extraanalytical Quality phase, Catlab, Viladecavalls, Barcelona, Spain; 14 Department of Laboratory Medicine, La Paz-Cantoblanco-Carlos III University Hospital, Madrid, Spain.

**Keywords:** glucose, preanalytical phase, glycolysis, diabetes mellitus, survey

## Abstract

**Introduction:**

Diabetes mellitus (DM) is one of the most prevalent diseases worldwide. The objective of this study was to find out under what preanalytical conditions routine and diagnostic glucose tests are performed across Spanish laboratories; and also what criteria are used for DM diagnosis.

**Materials and methods:**

An online survey was performed by the Commission on Quality Assurance in the Extra-Analytical Phase of the Spanish Society of Laboratory Medicine (SEQC-ML). Access to the questionnaire was available on the home page of the SEQC-ML website during the period April-July 2018. Data analysis was conducted with the IBM SPSS^©^ Statistics (version 20.0) program.

**Results:**

A total of 96 valid surveys were obtained. Most laboratories were in public ownership, serving hospital and primary care patients, with high and medium workloads, and a predominance of mixed routine-urgent glucose testing. Serum tubes were the most used for routine glucose analysis (92%) and DM diagnosis (54%); followed by lithium-heparin plasma tubes (62%), intended primarily for urgent glucose testing; point-of-care testing devices were used by 37%; and plasma tubes with a glycolysis inhibitor, mainly sodium fluoride, by 19%. Laboratories used the cut-off values and criteria recognized worldwide for DM diagnosis in adults and glucose-impaired tolerance, but diverged in terms of fasting plasma glucose and gestational DM criteria.

**Conclusion:**

Preanalytical processing of routine and DM diagnostic glucose testing in Spain does not allow a significant, non-quantified influence of glycolysis on the results to be ruled out. Possible adverse consequences include a delay in diagnosis and possible under-treatment.

## Introduction

Diabetes mellitus (DM) is one of the most prevalent diseases worldwide. According to the International Diabetes Federation (IDF), it had a prevalence of 451 million people in 2017, with a projection for 693 million by 2045 (*1*). In Spain, DM, mainly type 2 DM, is also one of the most prevalent diseases as reported in 2012 by the di@bet.es Study, establishing a prevalence of diabetes at 13.8%, which implies that about 4 million Spaniards were affected with type 2 DM (*2*).

Due to its high prevalence and associated complications, laboratory tests for its detection, control, and monitoring represent a significant workload. The most frequent test, requested in the vast majority of routine analyses, is fasting glucose concentration. Likewise, oral glucose tolerance tests (OGTT) continues to be in great demand for the diagnosis of gestational diabetes mellitus (GDM). Besides, the inclusion of glycated haemoglobin determination among the diagnostic criteria for DM, with greater preanalytical stability, has not replaced tests based on the determination of glucose concentration for several reasons: sensitivity, cost, test accessibility, presence of haemoglobinopathies, and non-applicability in GDM (*3*, *4*).

A preanalytical factor of great importance in glucose testing is glycolysis *ex vivo*. According to the recommendation of the World Health Organization (WHO) and the American National Academy of Clinical Biochemistry (NACB), the diagnostic sample must be venous plasma placed in ice-water slurry after collection, and separation from cells which needs to be conducted before 30 minutes (*5*, *6*). Otherwise, glycolysis will cause decreased glucose results with respect to their *in vivo* value.

Elimination of glycolysis is, therefore, essential in order to obtain reliable glycaemic results for control and diagnosis, since it introduces an unpredictable negative bias. Different additives may be used for this purpose. The most used is sodium fluoride (NaF) but, despite its widespread use, it is not currently recommended by international organizations (*7*, *8*). The recommended additive since 2011 is the citric-citrate buffer/NaF/ethylenediaminetetraacetic acid (EDTA) (*6*, *9*).

Implementation of the above-mentioned rules of sample management is not easy in laboratory routine (*10*). In addition, in our country, routine glycaemic testing is not usually carried out in plasma but in serum, as it allows the majority of tests of general biochemistry, immunochemistry, immunology, and serology to be performed. There is also no knowledge about the preanalytical treatment of glucose and DM diagnostic testing in Spanish laboratories since there are no references focused on this topic from a country perspective. This fact has motivated us to start this work.

The aim of this study was to find out under what preanalytical conditions routine and diagnostic glucose tests are carried out across Spain; and how they can affect the results according to the diagnostic criteria for DM. Our aim was also to verify the degree of adherence in our country to the recommendations of international organizations as well as to what extent this may influence the achievement of a quality diagnosis.

## Materials and methods

### Study design

An observational study consisting of an online survey was performed using Google Forms by the Commission on Quality Assurance in the Extra-Analytical Phase of the Spanish Society of Laboratory Medicine (SEQC-ML). The survey contained four sections: introduction; laboratory characteristics; tubes or devices used for glucose analyses; and, finally, DM diagnostic criteria used in the different laboratories. Access to the questionnaire was available on the home page of the SEQC-ML website during the period April-July 2018. Participation was voluntary and anonymous, and access was made available to partners by including it in the periodic news bulletins of the SEQC-ML during the aforementioned period.

Closed and mixed multiple-choice questions were used for the sections concerning laboratory characteristics and diagnostic criteria of DM. In the tubes and devices section, questions with Yes/No filter answers were applied, giving access to specific subsections with closed and mixed questions relating to preanalytical handling of each tube. All the questions from the different sections of the survey were to be answered by each laboratory in order to be included in the study. Omitted, contradictory or meaningless responses invalidated the survey or the specific section.

### Statistical analysis

Data analysis was performed with the IBM SPSS^©^ Statistics (v.20.0) program by frequency analysis. The results were obtained as frequencies and their percentages. In open-ended questions, counting was used and results are shown as percentages.

## Results

We obtained 105 responses from 700 laboratories associated with the SEQC-ML, with a response rate of 15%. Nine laboratories were dismissed: three of them did not report the tubes used, and six laboratories gave contradictory answers about the tube used and percentage or invalid answers. One laboratory was excluded only in DM diagnostic criteria section for incomplete response. A total of 96 valid surveys were obtained, with a confidence level of 95% and a margin of error of ± 9%.

The first five questions of the survey referred to the characteristics of the participating laboratories. Results are shown in Table 1.

**Table 1:** Laboratory characteristics

**Type of laboratory**	**Percentage**
Hospital	35
Hospital and primary care	61
Independent	4
Ownership	
Public	78
Private	14
State subsidised centre	8
Requests *per* year	
< 10,000	2
10,000 – 50,000	33
50,000 – 300,000	24
300,000 – 600,000	24
> 600,000	17
Glucose requests *per* day	
< 100	8
100 - 500	20
500 - 2000	29
> 2000	43
Sort of testing	
Routine	5
Urgent	2
Routine/urgent	93

The glycolysis inhibitor tube was used only by 19% of laboratories. Sodium fluoride was the glycolysis inhibitor used by 94% of laboratories. The serum tube was the most used for routine glucose tests. Only 21% of laboratories centrifuged serum samples in the first 30 minutes after blood collection and 25% centrifuged them between two and four hours after collection. The lithium-heparin tube (Li-Hep) was used for urgent glucose tests mainly (86%). Point of care testing (POCT), both whole blood sample in strips for glucometers and whole blood for blood gas analysers were used for two main purposes, urgent glucose tests and before OGTT. Serum and Li-Hep samples were kept mostly at room temperature before centrifugation.

Two different types of tubes/devices are used for glucose testing by 52% of the surveyed laboratories: 33% use both serum and Li-Hep and 12% both serum and POCT. Three types are used by 25% of laboratories, being Serum/Li-Hep/POCT the most frequent combination (18%). The serum tube was used as unique sample for glucose tests by 18% of participants, whereas 5% used the three types of tubes and POCT devices. Only 11% of laboratories included in their report a comment about the influence of glycolysis in the results when the precentrifugation time exceeded 30 minutes. These results are summarized in Table 2.

**Table 2:** Tubes and devices used

**Question 1:** Do you use a tube with glycolysis inhibitor in your laboratory for blood glucose testing?												
Yes: 19						No: 81						
**Question 2**: Do you use a serum tube with separating gel in your laboratory for blood glucose testing?												
Yes: 100						No: 0						
**Question 3:** Do you use a lithium heparin plasma tube in your laboratory for blood glucose testing?												
Yes: 62						No: 38						
**Question 4:** Do you use POCT devices for blood glucose testing? (equipment handled directly by laboratory staff)												
Yes: 37						No: 63						
**Question 5:** What percentage does this tube/device represents compared to the total of glucose tests done in your laboratory?												
Percentage	Glyc inhib				Serum				Li- Hep		POCT	
< 10%	67				3				24		86	
10 - 25%	17				1				59		11	
26 - 50%	11				1				12		3	
51 - 75%	5				8				2		0	
76- 90%	0				25				0		0	
> 90%	0				62				3		0	
**Question 6:** What do you use it for?*												
		Glyc inhib			Serum				Li- Hep		POCT	
Routine		0			30				5		6	
Urgent		4			18				86		37	
Diabetology		18			16				1		11	
Distant centers		22			19				0		4	
OGTT		56			17				8^†^		12	
Before OGTT		0			0				0		30	
**Question 7:** What type of glycolysis inhibitor additive do you use?												
Sodium Fluoride-Potassium Oxalate								94				
Citric/citrate buffer -fluoride-EDTA (liquid)								0				
Citric/citrate buffer -fluoride-EDTA (solid)								6				
Iodoacetate								0				
Other								0				
**Question 8:** How do you keep the sample from collection to centrifugation and analysis?												
				Glyc inhib			Serum					Li- Hep
In an ice-water slurry				0			0					5
Refrigerated				33			0					7
Room temperature				67			100					88
**Question 9:** How long does it take until centrifugation?												
			Serum							Li- Hep		
< 30 minutes in all cases			21							83		
< 2 hours in all cases			31							13		
< 2 hours mainly			23							2		
2-3 hours mainly			17							2		
2-4 hours mainly			8							0		
**Question 10:** If the collection-centrifugation period exceeds 30 minutes: Does the report include any warning mentioning the influence of glycolysis on glucose results?												
Yes: 11						No: 89						
**Question 11:** If the previous answer is affirmative, the origin of the warning is:												
Experimental study of the own laboratory						2						
Bibliographic references						7						
Other						1						
**Question 12:** What type of POCT device do you use?												
Glucometer with connectivity to LIS						0						
Glucometer without connectivity to LIS						64						
Blood gas analyser						36						
Other						0						
Results are presented as percentage. *Multiple answers are allowed: each laboratory uses the tube/device for several purposes. ^†^Immediate centrifugation after collection. Glyc inhib - glycolysis inhibitor plasma tube. Li-Hep - Lithium-heparin plasma tube. EDTA - ethylenediaminetetraacetic acid. POCT - Point of care testing. OGTT - Oral Glucose Tolerance Test. LIS – laboratory information system.												

Regarding the information about preanalytical issues of diagnostic tests and selected diagnostic criteria, the OGTT was performed by 87% of the laboratories at their own laboratory facilities and 75% of them declared centrifuging the tubes immediately after each collection, but the remaining 25% delayed centrifugation until the collection of the last sample.

The vast majority of the laboratories used cut-off values and criteria recognized worldwide, diverging with them in rare occasions. The most noticeable discrepancies occurred in the cut-off value of fasting blood glucose, reaching 16% of disagreement, and in GDM diagnostic criteria. Questions and results are shown in Table 3.

**Table 3:** Information about diagnostic tests

**Question 1:** Where do you perform OGTT?	
At the laboratory	68
At peripheral facilities	9
GDM Screening at peripheral facilities, OGTT at the laboratory	19
Other	4
**Question 2:** Treatment of OGTT samples:	
Centrifugation after each collection, analysis	75
RT, centrifugation after last collection, analysis	20
Refrigerated, centrifugation after the last collection, analysis	5
Other	0
**Question 3:** What reference values do you use for routine fasting glucose test in adults?	
3.9 - 6.05 mmol/L, WHO; 1999	50
3.9 - 5.6 mmol/L, ADA; 2003	34
Other	16
**Question 4:** What cut-off value do you use in OGTT to diagnose DM in adults?	
Fast 8h/OGTT 75g/Glucose 2h 11.1 mmol/L	100
Other	0
**Question 5:** What cut-off value do you use in OGTT for impaired glucose tolerance?	
Fast 8h/OGTT 75g/Glucose 2h 11.1 mmol/L	98
Other	2
**Question 6:** What cut-offs value do you use to diagnose GDM?	
ONE STEP: 24-28 week, fast 8h/OGTT 75g/ Basal 7.0 mmol/L; 2h 7.8 mmol/L, WHO; 1999	12
ONE STEP: 24-28 week, fast 8h/ OGTT 75g/ Basal 5.1 mmol/L; 1h 10.0 mmol/L; 2h 8.5 mmol/L, IADPSG; 2010, ADA; 2016	10
TWO STEPS: 24-28 week. *STEP 1:* O’Sullivan test OGO 50g/ 1h glucose 7.8 mmol/L; *STEP 2:* Fast 8h/OGTT 100g/ Basal 5.8 mmol/L; 1h 10.6 mmol/L; 2h 9.2 mmol/L; 3h 8.0 mmol/L, NDDG; 1979, ADA; 2016, GEDE; 2006	56
TWO STEPS: *STEP 1:* O’Sullivan OGO 50g/ 1h 7.8 mmol/L; *STEP 2:* fast 8h/OGTT 100 g/ Basal 5.3 mmol/L; 1h 10.0 mmol/L; 2h 8.6 mmol/L; 3h 7.8 mmol/L, Carpenter/Coustan; 1982, ADA; 2016	12
Other*	10
Results are presented as percentage. *Laboratories using two criteria of the previous ones: 4%. Only O’Sullivan test 6%. WHO - World Health Organization. ADA - American Diabetes Association. OGTT - Oral Glucose Tolerance Test. NDDG - National Diabetes Data Group. GEDE - Spanish Group of Diabetes and Pregnancy. OGO - Oral glucose overload. IADPSG - International Association of Diabetes and Pregnancy Study Group.	

## Discussion

To the best of our knowledge, only one previous manuscript described a similar study, focused on a country perspective, in Croatia (*11*). With this survey we confirmed that the serum tube was the most frequent for routine glucose tests, including those from peripheral collection centres, tests requested by emergency rooms and even for OGTT and tests requested for diabetologists, in accordance with the data reported by Vučić *et al*., although in this study there was no specific mention of the diagnostic tests of DM (*11*). These data suggest an influence of the *in vitro* glycolysis impossible to quantify in the majority of routine glucose tests. According to scientific evidence, the decrease with respect to the *in vivo* value is because centrifugation of the serum tube can never be performed immediately after blood collection, but rather after completing the formation of a stable clot, which can take from 10 minutes to half an hour depending on the tube (*12*).

The issue is aggravated when centrifugation is delayed or the sample is kept at room temperature. From our survey results, this delay exceeds two hours, even three, in many occasions, as occurs in serum samples of peripheral collection centres without prior centrifugation and kept mostly at room temperature. Slightly shorter delays are mentioned in the study conducted in Croatia in which 34% of samples were centrifuged before 30 minutes, 57% before two hours, and 9% after more than two hours (*11*). Moreover, the widespread use of serum tubes does not meet the conditions of fasting plasma glucose diagnostic criterion (*13*, *14*). One explanation of the predominant use of serum tubes for routine glucose analysis could be that the serum sample is useful for the majority of biochemistry tests. The cost of introducing new tubes and the need to change work routines could also have an influence.

Oral glucose tolerance test was mainly performed in the serum tube and many laboratories did not centrifuge the samples until the last collection. This is more dramatic in OGTT for GDM diagnosis, because the last tube is collected at three hours, according to 68% of the laboratories.

The tube with glycolysis inhibitor was used by a minority of the surveyed laboratories and the most frequent was NaF. The study carried out in Croatia reported a similar use of this type of tubes without specifying the inhibitor (*11*).

The most effective glycolysis inhibitor, buffer citric-citrate/NaF/EDTA is recommended by the American Diabetes Association (ADA) but is used by only one surveyed laboratory (*6*). In these recommendations, the use of NaF is discouraged because, according to evidences, it does not stop glycolysis up to four hours after blood collection (*15*, *16*). In addition, NaF produces an increase of haemolysis up to in 50% of samples (*16*). Nevertheless, in Spain continues to be predominant.

The first lyophilized citric-citrate buffer additive was replaced by a liquid additive that required the use of a controversial dilution factor (*17*-*19*). But, since 2017, it has been replaced by a validated lyophilized additive (*16*, *20*). Slightly higher plasma glucose results are also observed with this additive than with the WHO standard, although Van der Bergh *et al*. verified that, even in these supposedly ideal conditions, glycolysis occurs (*21*).

Fasting plasma glucose test is included as criterion for detection of pre-diabetes and DM in the clinical guidelines of the Spanish Diabetes Society, in which only a brief mention of the pre-analytical drawbacks is made. An alert in laboratory reports about the probable influence of *in vitro* glycolysis could be advisable (*22*).

Regarding GDM diagnostic values, most laboratories preferred the National Diabetes Data Group (NDDG) 1979 two-step criterion, recommended by the Spanish Society of Gynaecology and Obstetrics (SEGO), with cut-off values lower than the International Association of Diabetes and Pregnancy Study Groups (IADPSG) and Hyperglycaemia and Adverse Pregnancy Outcome (HAPO) criterion, the most recent one in which the cut-off values were obtained by taking special care in reducing glycolysis (*23*-*26*). According to the results of a survey conducted by the Spanish Society of Endocrinology and Nutrition (SEEN) in 2018 among its associates, 43% of them rejected the change to the HAPO criterion for not reporting benefits to the Spanish population and 35% was favourable to change, but accompanied by strategies and resources that allow proper attention (*27*).

One limitation of this study is the low response rate, but we believe it provides interesting preliminary data. We assume a single response *per* laboratory as requested in the survey.

Preanalytical conditions of the routine analysis of fasting glucose level in Spain do not allow ruling out a significant, non-quantified influence of *in vitro* glycolysis on the results obtained, and physicians are not informed about this influence. The conditions in which oral glucose tolerance tests are performed do not conform to the ideal ones due to the widespread use of serum for this purpose. Possible adverse consequences include a delay in the diagnosis of diabetes or pre-diabetes and possible follow-up under-treatment.

It is mandatory a pedagogical effort by national scientific societies to improve awareness of laboratory professionals and clinicians about this problem. Steps should be taken encouraging the use of more efficient glycolysis inhibitor additives, such as a change of the tubes for sample collection. Although this could involve higher costs in the short term to the laboratory, it would lead to higher quality of diagnostic tests and a benefit for patients, saving costs in the long term.

Changes in the cut-off values of diagnostic tests should also be considered, in order to adapt them to the elimination of *in vitro* glycolysis effect.
