# Corrigendum to: Preanalytical issues related to routine and diagnostic glucose tests: Results from a survey in Spain

**DOI:** 10.11613/BM.2020.021201

**Published:** 2020-04-15

**Authors:** Isabel García-del-Pino, Josep M. Bauça, Carolina Gómez, Andrea Caballero, María Antonia Llopis, Mercedes Ibarz, Débora Martínez, Montserrat Ventura, Itziar Marzana, Juan J. Puente, Marta Segovia, Paloma Salas, Rubén Gómez-Rioja

**Affiliations:** 1 Spanish Society of Laboratory Medicine, Extra-analytical Quality Commission, Barcelona, Spain; 2 Area Laboratory, A Coruña University Hospital Complex, A Coruña, Spain; 3 Department of Laboratory Medicine, Son Espases University Hospital, Palma, Balearic Islands, Spain; 4 Department of Clinical Analysis and Biochemistry, Laboratori Clínic Metropolitana Nord, Germans Trias i Pujol Hospital, Badalona, Spain; 5 Preanalytic Area, Department of Clinical Biochemistry, Vall d’Hebron Hospital, Barcelona, Spain; 6 Clinical Laboratories Corporate Coordination, Catalan Health Institute, Barcelona, Spain; 7 Department of Laboratory Medicine, Arnau de Vilanova University Hospital, IRBLleida, Lleida, Spain; 8 Department of Laboratory Medicine, University of Navarra Clinic, Madrid, Spain; 9 External Quality Assurance Programmes, Spanish Society of Laboratory Medicine, Barcelona, Spain; 10 Department of Laboratory, Cruces University Hospital, Barakaldo, Bizkaia, Spain; 11 Department of Biochemistry, ‘Lozano Blesa’ University Clinical Hospital, Zaragoza, Spain; 12 Department of Laboratory Medicine, La Paz-Cantoblanco-Carlos III University Hospital, Madrid, Spain; 13 Department manager of Preanalytical and Extraanalytical Quality phase, Catlab, Viladecavalls, Barcelona, Spain; 14 Department of Laboratory Medicine, La Paz-Cantoblanco-Carlos III University Hospital, Madrid, Spain.

This is a correction of Biochem Med (Zagreb) 2020;30(1):010704. DOI: https://doi.org/10.11613/ BM.2020.010704

Since the publication of the article, the authors have noticed an error in Table 3, Question 5. The correct Table is presented below. The authors apologize for any inconvenience caused to the readers.

**Table 3:** Information about diagnostic tests

**Question 1:** Where do you perform OGTT?	
At the laboratory	68
At peripheral facilities	9
GDM Screening at peripheral facilities, OGTT at the laboratory	19
Other	4
**Question 2:** Treatment of OGTT samples:	
Centrifugation after each collection, analysis	75
RT, centrifugation after last collection, analysis	20
Refrigerated, centrifugation after the last collection, analysis	5
Other	0
**Question 3:** What reference values do you use for routine fasting glucose test in adults?	
3.9 - 6.05 mmol/L, WHO; 1999	50
3.9 - 5.6 mmol/L, ADA; 2003	34
Other	16
**Question 4:** What cut-off value do you use in OGTT to diagnose DM in adults?	
Fast 8h/OGTT 75g/Glucose 2h 11.1 mmol/L	100
Other	0
**Question 5:** What cut-off value do you use in OGTT for impaired glucose tolerance?	
Fast 8h/OGTT 75g/Glucose 2h 7.8 -11.1 mmol/L	98
Other	2
**Question 6:** What cut-offs value do you use to diagnose GDM?	
ONE STEP: 24-28 week, fast 8h/OGTT 75g/ Basal 7.0 mmol/L; 2h 7.8 mmol/L, WHO; 1999	12
ONE STEP: 24-28 week, fast 8h/ OGTT 75g/ Basal 5.1 mmol/L; 1h 10.0 mmol/L; 2h 8.5 mmol/L, IADPSG;2010, ADA; 2016	10
TWO STEPS: 24-28 week. *STEP 1:* O’Sullivan test OGO 50g/ 1h glucose 7.8 mmol/L; *STEP 2:* Fast 8h/OGTT 100g/ Basal 5.8 mmol/L; 1h 10.6 mmol/L; 2h 9.2 mmol/L; 3h 8.0 mmol/L, NDDG; 1979, ADA; 2016, GEDE;2006	56
TWO STEPS: *STEP 1:* O’Sullivan OGO 50g/ 1h 7.8 mmol/L; *STEP 2:* fast 8h/OGTT 100 g/ Basal 5.3 mmol/L; 1h 10.0 mmol/L; 2h 8.6 mmol/L; 3h 7.8 mmol/L, Carpenter/Coustan; 1982, ADA; 2016	12
Other*	10
Results are presented as percentage. *Laboratories using two criteria of the previous ones: 4%. Only O’Sullivan test 6%. WHO - World Health Organization. ADA - American Diabetes Association. OGTT - Oral Glucose Tolerance Test. NDDG - National Diabetes Data Group. GEDE - Spanish Group of Diabetes and Pregnancy. OGO - Oral glucose overload. IADPSG - International Association of Diabetes and Pregnancy Study Group.	

